# Supplementary material for: Design and evaluation of a prototype medical device for robotic and manual percutaneous dilatational tracheostomy
Source: PLoS One. 2026 Feb 27;21(2):e0343014. doi: 10.1371/journal.pone.0343014 (PMC12948056; doi:10.1371/journal.pone.0343014)
Supplement: S1 File — (PDF) [file pone.0343014.s001.pdf]

Research Governance, Ethics and Integrity

2nd Floor Christie Building

The University of Manchester

Oxford Road

Manchester

M13 9PL

Email: research.ethics@manchester.ac.uk

Ref: 2024-23611-41684

23/05/2024

Dear Yuan Tang, Dr Andrew Weightman ,

Study Title: Design and evaluation of a prototype medical device for robotic and manual percutaneous dilatational tracheostomy

Project start date: 01/06/2024

We hereby confirm that you have used the University of Manchester's Ethics Decision Tool for the above study and provided further information and project details in the Ethics Review Manager (ERM) system. Based on the information you have provided, the conclusion is that **formal ethical review is not necessary** in accordance with University policy and the study is classed as ethically exempt.

Please note that you are still required to adhere to all University expectations and requirements in relation to the conduct of your project, including ethics best practice. You are also required to adhere to all University data protection policies and procedures.

If for any reason the details and specifications of your research need to be updated or refined you will need to re-visit the University Ethics Decision Tool and associated guidance to determine if the study remains ethically exempt.

We wish you every success with the project.

Yours sincerely,
Research Ethics Team
