# Supplementary material for: Design and evaluation of a prototype medical device for robotic and manual percutaneous dilatational tracheostomy
Source: PLoS One. 2026 Feb 27;21(2):e0343014. doi: 10.1371/journal.pone.0343014 (PMC12948056; doi:10.1371/journal.pone.0343014)
Supplement: Attachment — Submitted filename: Response letter.docx [file pone.0343014.s003.docx]

We thank both reviewers and the editors for the time taken to provide thoughtful reviews of our manuscript. We have responded to reviewers' comments to the best of our abilities, and we hope the paper is now acceptable for publication. We have added our responses in blue, and the manuscript sections with grey highlights (with blue representing new text). We have also summarised the amendments made to relevant sections of the manuscript.

**Journal Requirements:**

**1. When submitting your revision, we need you to address these additional requirements.**

**Please ensure that your manuscript meets PLOS ONE's style requirements, including those for file naming. The PLOS ONE style templates can be found at**

[**https://journals.plos.org/plosone/s/file?id=wjVg/PLOSOne_formatting_sample_main_body.pdf**](https://journals.plos.org/plosone/s/file?id=wjVg/PLOSOne_formatting_sample_main_body.pdf) **and**

[**https://journals.plos.org/plosone/s/file?id=ba62/PLOSOne_formatting_sample_title_authors_affiliations.pdf**](https://journals.plos.org/plosone/s/file?id=ba62/PLOSOne_formatting_sample_title_authors_affiliations.pdf)

Thank you for the helpful comment. The manuscript has been reformatted according to the journal requirements.

**2. Please update your submission to use the PLOS LaTeX template. The template and more information on our requirements for LaTeX submissions can be found at** [**http://journals.plos.org/plosone/s/latex**](http://journals.plos.org/plosone/s/latex)**.**

Thank you for the helpful comment. The manuscript has been reformatted according to the journal requirements.

**3. We are unable to open your Supporting Information file “Trachy data2.sav”. Please kindly revise as necessary and re-upload.**

Thank you for the helpful comment. The supplement materials have been reformatted.

**4. Your ethics statement should only appear in the Methods section of your manuscript. If your ethics statement is written in any section besides the Methods, please move it to the Methods section and delete it from any other section. Please ensure that your ethics statement is included in your manuscript, as the ethics statement entered into the online submission form will not be published alongside your manuscript.**

Thank you for the helpful comment. The ethics section has been reformatted.

**5. Please include captions for your Supporting Information files at the end of your manuscript, and update any in-text citations to match accordingly. Please see our Supporting Information guidelines for more information:** [**http://journals.plos.org/plosone/s/supporting-information**](http://journals.plos.org/plosone/s/supporting-information)**.**

Thank you for the helpful comment. The supplement materials have been reformatted.

**6. If the reviewer comments include a recommendation to cite specific previously published works, please review and evaluate these publications to determine whether they are relevant and should be cited. There is no requirement to cite these works unless the editor has indicated otherwise.** 

Thank you for the helpful comment. We have addressed the reviewers’ comments accordingly. The details are shown below.

**Reviewer #1: This manuscript is highly creative and ambitious, offering content that is both engaging and thought-provoking.
With minor refinements in readability and stricter adherence to the journal’s submission guidelines, it has the potential to become an outstanding contribution.
I would like to praise this study for its challenging scope and engaging nature.**

Thank you very much for the comment. We have double-checked the full text and refined the writing. We have also reformatted the manuscript according to the journal requirements.

**Reviewer #2: The work is very interesting and is the need of hour. The present research is original in terms of the dilator design and mechanism. A qualitative performance comparison of the dilator design with that of the existing PDT device was done and presented and appears satisfactorily and is novel.
Comparison of the mean insertion force peak with that of the other device available in the market shows the reduction in the force which is a good pointer to pitch for this device though the reduction is not significant. Notwithstanding this the proposed PDT device may provide further insights for future improvement in the device’s design in reducing the pain further and negating other procedural difficulties.
The article complies with the standards of the journal and also in terms of reporting guidelines etc. The content, results and discussion, analyses, presentation and language are good.
Following suggestions may help in the completeness of the manuscript:
1. Uncertainty in the obtained results be presented for better understanding of the performance of the device.**

Thank you very much for the comment. We have added more explanations to the discussion section to explain the uncertainty of the obtained results. The modified text is:

The uncertainty of the results obtained from the experiments was dominated by the material properties of the porcine tissue, which could be affected by the meat cut, thickness, and freshness. No significant deformation of the TrachyPen parts was observed, nor was there a significant deviation from the defined movement trajectory. The uncertainty could be mitigated by selecting the samples from the same fresh porcine back cut with consistent thickness.
